# Pinopsin Regulates Melatonin Production and Daily Locomotor Activity: Functional Insights From Gene‐Edited *Xenopus* Tadpoles

**DOI:** 10.1111/jpi.70114

**Published:** 2026-01-27

**Authors:** Neda Heshami, Ricardo D. Romero, Flavio S. J. de Souza, Gabriel E. Bertolesi, Sarah McFarlane

**Affiliations:** ^1^ Department of Cell Biology and Anatomy Hotchkiss Brain Institute Calgary Alberta Canada; ^2^ Department of Cell Biology and Anatomy Alberta Children's Hospital Research Institute Calgary Alberta Canada; ^3^ Biología Molecular y Neurociencias (IFIBYNE‐UBA‐CONICET) Instituto de Fisiología Ciudad de Buenos Aires Argentina; ^4^ Departamento de Fisiología, Biología Molecular y Celular, (FBMC‐FCEyN‐UBA), Facultad de Ciencias Exactas y Naturales Universidad de Buenos Aires Ciudad de Buenos Aires Argentina

**Keywords:** chronobiology, circadian rhythm, frogs, light sensitivity, molecular evolution, pigmentation, Pineal gland

## Abstract

Circadian rhythm alignment depends on environmental light detection via opsins. Pinopsin, originally identified in the pineal organ of birds and later in amphibian pineal complex and eyes, may play a role in this process, though its function has not been genetically tested. Evolutionary analysis suggests pinopsin was independently lost in several vertebrate lineages, including mammals (Synapsida), some reptiles (e.g. snakes and crocodiles), and teleost fish, but retained in birds, turtles, lizards, and non‐teleost Actinopterygii. We conducted a detailed genomic search of the pinopsin gene across 95 amphibian species and assessed its function in *Xenopus laevis* tadpoles using CRISPR/Cas9‐mediated knockout. Our survey indicates that pinopsin is highly conserved in salamanders and most anurans, but absent in many caecilians (Gymnophiona), which have a fossorial lifestyle with limited light exposure. To investigate its biological role, we generated *X. laevis* F0 pinopsin knockout tadpoles and evaluated two light‐sensitive responses: (1) day/night melatonin fluctuations inferred from skin pigmentation changes, and (2) locomotor activity over a 24‐h photoperiod. We show these responses depend only on pineal light sensitivity and are independent of eye sensitivity at developmental stage 46/47. Our findings reveal: (1) Pinopsin is co‐expressed with Aanat, a key enzyme in melatonin synthesis; (2) knockout tadpoles show paler skin during the light phase, suggesting pinopsin suppresses melatonin production in daylight; and (3) reduced daytime locomotor activity in F0 mutants, consistent with melatonin‐induced lethargy. Overall, pinopsin emerges as a critical opsin for light‐regulated circadian‐associated behavior in *Xenopus*, with likely conserved roles across amphibians (anurans and salamanders in general) and other non‐mammalian vertebrates, including birds, turtles, and lizards.

## 1 Introduction

Vertebrates align their internal circadian clocks with day‐night cycles using opsins, light‐sensitive molecules that link environmental light to biological rhythms like sleep and melatonin secretion. Most insights into opsin‐mediated photoentrainment come from mammals, where melanopsin‐expressing retinal cells signal to the brain's master clock, the suprachiasmatic nucleus, to regulate circadian rhythms, sleep latency, and melatonin production [3, 4, 5, 6, 7]. In contrast, the role of photosensitivity in circadian regulation among non‐mammalian vertebrates remains less understood. This gap in knowledge stems in part from a mammalian‐centric focus on ocular light detection, which overlooks the pineal organ. A nocturnal phase in mammalian evolution likely contributed to the loss of pineal photosensitivity, further limiting our understanding of extraocular photoreception in other vertebrate lineages [8]. Non‐mammalian vertebrates retained a light‐sensitive pineal complex that expresses unique opsins, including pinopsin that was first identified in chickens [9] and may act as an extraocular photosensing protein. Recent evidence suggests that pinopsin is also expressed in the eye and may contribute to dim‐light vision [10]. The precise biological role of pinopsin remains unclear and has not yet been genetically tested.

In non‐mammalian vertebrates, the pineal complex is located dorsally on the head and contains light‐sensitive opsins. Here, we use the term pineal complex to refer collectively to the pineal gland and its accessory structures, which vary in nomenclature and morphology across clades. These accessory structures include the frontal organ in amphibians, the parietal eye in the tuatara and many lizards, and the parapineal organ in lampreys and fish [11]. Notably, while accessory organs are absent in birds, their pineal complex remains photosensitive [12]. In contrast, the pineal gland in mammals lost its light sensitivity and was structurally incorporated into the brain during evolution [8, 11, 12]. Opsin activation provides an efficient mechanism for synchronizing the internal circadian clock with environmental light‐dark cycles. Melatonin, an ancient hormone, is secreted by the pineal complex under tight regulation, regardless of whether the organism is diurnal or nocturnal [13, 14]. In non‐mammalian vertebrates this regulation occurs via pineal complex photosensitivity, whereas in mammals it is mediated by ocular photoreception [14]. Light detected by opsins inhibits melatonin synthesis, which otherwise occurs in darkness. In mammals, melanopsin (OPN4) in the eye plays a central role in transducing environmental light into circadian regulation [15]. Non‐mammalian vertebrates, however, express a broad repertoire of opsins in the pineal complex, including pinopsin (*opnp*), parapinopsin (*opnpp*), parietopsin (*opnpt*), and vertebrate ancient opsin (*VA‐opsin*), all of which were lost during mammalian evolution [12, 16, 17]. Melanopsin (OPN4) is the most well‐characterized opsin involved in circadian physiology in mammals. Interestingly, the *opn4* gene is duplicated in some non‐mammalian vertebrates (*opn4* and *opn4b*; also referred to as *opn4m* for mammalian and *opn4x* for *Xenopus*) and is expressed in the pineal complex of amphibians, reptiles, and birds [2, 18, 19, 20]. Despite this diversity, the primary opsin responsible for regulating melatonin production and circadian rhythms in non‐mammalian vertebrates remains unknown.

We focus here on pinopsin, an opsin found in the pineal complex and eyes of some vertebrates [10, 21]. Pinopsin's presence in extraocular tissue and blue‐light sensitivity (∼470 nm), crucial for circadian regulation, suggest a role in melatonin synthesis [22]. Interestingly, recent studies identified pinopsin in the eyes of non‐teleost fish and amphibians [2, 10, 21]. Biochemical analyses of spotted gar and frog pinopsin confirmed its blue‐light sensitivity and thermal isomerization similar to rhodopsin, implying a possible function in dim‐light vision [10]. Studies indicate pinopsin was independently lost many times during vertebrate evolution including lampreys, teleosts and again in mammals, as well as in some reptile clades like crocodiles and snakes [16, 23, 24, 25, 26]. Pinopsin expression in both the eye and the pineal complex is intriguing. However, its function as a visual or extraocular opsin remains genetically untested, and a comprehensive evolutionary analysis across amphibian lineages is still lacking.

Plasma melatonin levels reflect the duration of the light‐dark period and are highest in darkness [15]. The rate‐limiting enzyme of melatonin biosynthesis in the pineal complex is aralkylamine N‐acetyltransferase (Aanat), whose activity is synchronized by environmental light signals [15, 27, 28]. In all studied vertebrates, the light‐dark cycle controls the expression of the circadian clock genes and downstream Aanat expression and activation, thus controlling the rhythm of melatonin production [11, 15]. Interestingly, in amphibian *Xenopus laevis* tadpoles the variation in skin colour (darkness/lightness) provides an easy read‐out for the plasma levels of melatonin that fluctuate across the light‐dark cycle [29, 30]. Pigmented melanophores in the skin contain melanosomes filled with a black pigment melanin. The melanosomes aggregate in the presence of melatonin to make the skin lighter at night [17, 30, 31]. When melatonin levels drop in the light phase, melanosomes disperse, which leads to darker skin [30]. Thus, skin color changes in tadpoles are a useful readout of melatonin levels.

In this study, we performed an evolutionary analysis of pinopsin and explored its biological function. We demonstrated pinospin is co‐expressed with Aanat in melatonin‐synthesizing cells. Using CRISPR/Cas9 gene editing we observed that pinopsin in the pineal complex regulates the daily locomotor activity and melatonin production. Specifically, pinopsin F0 CRISPR mutants show impaired light‐induced melatonin suppression, as revealed by the skin pigmentation assay, and less motor activity in light as compared to wild type larvae. Overall, our findings highlight pinopsin as a key opsin in several non‐mammalian vertebrates for the regulation of light/dark driven behaviors.

## 2 Materials and Methods

### 2.1 Evolutionary Analyses

Genomes of Lissamphibia species deposited in the NCBI Genome database (https://www.ncbi.nlm.nih.gov/datasets/genome/) were searched with the 345‐amino acid sequence of *X. tropicalis* pinopsin (XP_002934391) using the TBLASTN program. A complete list of species and genome versions analyzed, as well as the deduced amino acid sequences of all pinopsin proteins retrieved are shown in Supporting Information Table S1. For species with well‐assembled genomes containing large scaffolds, the conservation of the neighbouring genes of pinopsin was also ascertained with the help of the MultiPipMaker [32]. Species lacking pinopsin altogether or carrying probable inactivating mutations in the gene are shown in Supporting Information Table S2.

A phylogenetic tree of pinopsin proteins was built with the NGPhylogeny program package [https://ngphylogeny.fr/ [33]]. Sequences were aligned with Clustal Omega and ambiguous regions trimmed with Gblocks [34], resulting in a final alignment of 332 residues. A maximum likelihood tree was built with the PhyML 3.0 program [35] using the JTT model of amino acid substitution, with four categories of substitution rates across sites and estimated gamma parameter, amino acid frequencies, and invariable sites (+G+I+F). Tree refinement was done by the Subtree Pruning and Regrafting (SPR) method, and statistical robustness was evaluated with a fast likelihood ratio test (SH‐like aLRT [35]). The evolutionary relationship between amphibian clades was based on previous studies [1, 36].

### 2.2 *Xenopus laevis* Embryos Fertilization, Surgery, and Light/Dark Conditions

All procedures involving frogs and embryos were approved by the Animal Care and Use Committee at the University of Calgary. Embryos were obtained via induced ovulation in females injected with chorionic gonadotrophin (Intervet Canada Ltd.), followed by *in vitro* fertilization using standard protocols. Detailed procedures for fertilization and developmental staging are available on Xenbase (http://www.xenbase.org/, RRID: SCR_003280). Embryos were maintained at 16°C for the first 48hours, then raised at 20°C–24°C until the desired developmental stage. They were kept in Marc's Modified Ringer (MMR; 100 mM NaCl, 2 mM KCl, 2 mM CaCl₂, 1 mM MgCl₂, 5 mM HEPES, pH 7.4) under a 12‐h light/12‐h dark cycle (light intensity ~1000 lux or 1.5 × 10⁻⁴ W/cm²). Note that embryos at stage 46/47 or younger cannot be sexed. Enucleation and mock surgeries were performed on stage 42/43 tadpoles. Behavioral studies were conducted 48hours post‐surgery, when tadpoles reached stage 46/47.

### 2.3 Determination of the Pigmentation Index

Skin pigmentation changes were quantified using a pigmentation index, as previously described [37]. Briefly, dorsal head images of tadpoles were captured under consistent lighting and exposure conditions using a stereoscope (Stemi SV11; Carl Zeiss Canada Ltd.) and a digital camera (Zeiss Axiocam HRC). Images were converted to binary black‐and‐white format using NIH ImageJ (U.S. National Institutes of Health, Bethesda, MD). Representative pre‐conversion images are shown in Figure 2A. Positive pixel over the head areas from binary images were quantified and analyzed using NIH ImageJ software. Statistical comparisons were performed using the GraphPad Prism 10.1 software as described below.

### 2.4 Daily Locomotor Behavior Assays

Stage 46/47 tadpoles were individually housed in wells of 12‐well plates and habituated prior to behavioral testing. Locomotor activity was recorded over a 24‐h period using the ZebraBox system (Viewpoint Life Sciences) under a 12‐h light/12‐h dark cycle. Movement was tracked in 30‐min intervals using Viewpoint Life Sciences software, which calculated the distance traveled and corresponding movement speed. Locomotor activity levels during light and dark phases were quantified for each tadpole and compared across experimental groups, including wild type, enucleated, and mutant embryos. At the end of the experiment, dead tadpoles with no movement response or malformations were removed from analysis.

### 2.5 Immunohistochemistry in Whole Embryos and Tissue Sections

A polyclonal antibody specific to the C‐terminus of *Xenopus* pinopsin was custom‐generated (Pacific Immunology Corp., USA, CA) in goat against a synthetic 16‐amino acid peptide (CRTDVTSVSEAGGNKV) conjugated to keyhole limpet hemocyanin. The antibody was produced and affinity‐purified by Pacific Immunology Corp. (Project Report PAC‐17147 goat) following their standard protocols. Whole‐mount and section immunohistochemistry were performed as described previously [2]. Embryos were fixed overnight in 4% paraformaldehyde at 4°C. To enhance antibody penetration, two small incisions were made in the skin over the brain on both sides of the head. Whole embryos were incubated overnight at 4°C with the anti‐pinopsin antibody (1:100) in PBT buffer [PBS containing 0.2% bovine serum albumin (Sigma, St. Louis, USA), 0.1% Triton X‐100 (Sigma), and 5% donkey serum]. After rinsing in PBT, samples were incubated with an Alexa Fluor 488‐conjugated anti‐goat secondary antibody (1:500; Thermo‐Fisher) at 4°C. Fluorescent images of dorsal heads from wild type and F0 tadpoles were captured using a fluorescence stereoscope to assess pinopsin knockdown. Fluorescence intensity was quantified using NIH ImageJ software and correlated quantitatively with skin pigmentation indices. Neurofilament‐Associated Antigen (NAA) immunohistochemistry in whole mount tadpoles used the same protocol, via mAb 3A10, (1:50 dilution; Developmental Studies Hybridoma Bank; Iowa, USA), followed by an Alexa Fluor 647‐conjugated anti‐rabbit secondary antibody. For section immunostaining, 12 μm cryosections were prepared with tadpoles embedded in Optimal Cutting Temperature medium (OCT; Tissue TEK). The same immunostaining protocol was applied, with reduced incubation times (1h) and primary and secondary antibody dilution (1:200 and 1:1000, respectively). To detect AANAT‐expressing cells, a rabbit anti‐AANAT antibody [ab3505; (Abcam), dilution 1:100] was used in combination with an Alexa Fluor 647‐conjugated anti‐rabbit secondary antibody.

### 2.6 Generation and Validation of F0 Pinopsin Knockdown in *Xenopus laevis* Tadpoles

Single guide RNAs (sgRNAs) targeting exon 1 of the pinopsin gene were designed using CHOPCHOP CRISPR design tool (https://chopchop.cbu.uib.no/). The selected sgRNA sequences were: sgRNA1: ATCTTAGCTTCTTGTACTTA and sgRNA2: GTACTGCGAGGGGCTAGATG. To generate F0 CRISPR/Cas9 mutants, 1 nL of each sgRNA was co‐injected with recombinant Cas9 protein (Integrated DNA Technology; Coralville, Iowa; USA) into single cell *Xenopus* embryos. Microinjections were performed by using glass microelectrodes within the first hour post‐fertilization. Knockdown validation was conducted via whole‐mount immunohistochemistry on stage 45/46 larval tadpoles or at the end of each experiment.

### 2.7 Microscopy

Images of fixed embryos were taken with an Axio‐Cam HRc (Carl Zeiss) on a Stemi SVII stereomicroscope (Carl Zeiss). Z‐stack images of section immunohistochemistry were taken with a LSM900 Zeiss Confocal microscope and the ZEN blue system which were processed for brightness and contrast with Adobe Photoshop. The 3D image structure of Neurofilament‐Associated Antigen and pinopsin immunostaining in the pineal complex was generated with IMARIS software (Oxford Instruments, UK) using the surface tool to classify each stained segment.

### 2.8 Statistical Analysis

Statistical analyses were performed by using Prism 10 (GraphPad). One‐way ANOVA followed by Tukey's post hoc test was applied to assess significance across multiple treatment groups, while unpaired t‐tests with Welch's correction were used for comparisons between two groups. Locomotor activity, measured as traveled distance in 30‐min intervals, was visualized using heatmaps generated in Python, scaled from 0 to 255. Pearson's correlation coefficient (r) was used to evaluate the association between pigmentation index and pinopsin expression, as determined by fluorescence intensity.

## 3 Results

### 3.1 The Pinopsin Gene was Occasionally Lost During Evolution but is Present in a Majority of Anuran Genomes

**Figure 1:**
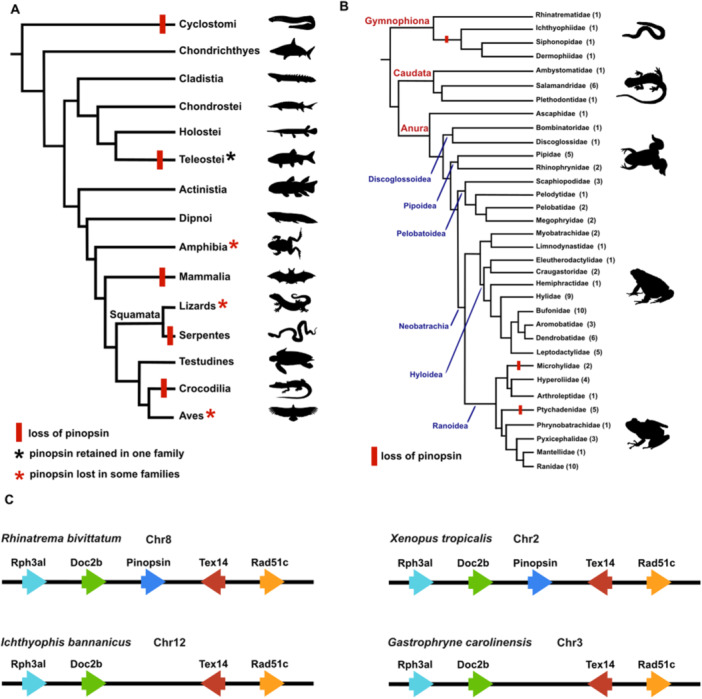
Pinopsin was lost sporadically in vertebrates but is largely conserved in extant Amphibians. (A) Phylogenetic tree of vertebrates indicating the loss of the pinopsin gene in different vertebrates lineages (red bar), based on partial evolutionary analysis from various authors [16, 23, 24, 25, 26, 39]. (B) Phylogenetic distribution of pinopsin within Amphibia shown at the family level. The number of species surveyed for each family is shown in parentheses. A total of 95 amphibian species from 34 families were analysed. Red bars indicate that pinopsin could not be retrieved from species from that branch. The tree topology is consistent with previously reported phylogenetic relationships [1, 36]. (C) Schematics of the pinopsin chromosomal loci in species of Gymnophiona (*R. bivitattum*, *I. banannicus*) and Anura (*X. tropicalis*, *G. carolinensis*), showing synteny conservation and lack of pinopsin in two species. An expanded version of this figure is shown in Figure S2.

Pinopsin is an ancient vertebrate gene detected in the pineal gland of several non‐mammalian vertebrates [9, 10, 38]. Pinopsin exhibits a complex evolutionary history marked by multiple independent losses [10, 24, 25, 26]. The structural reorganization of the pineal complex across vertebrate lineages appears to correlate, potentially causally, with inactivation of the pinopsin gene. For instance, pinopsin loss in crocodilians and mammals is likely attributed to the absence of a functional pineal organ in the former, and to the organ's incorporation deep within the brain, resulting in loss of superficial light sensitivity, in the latter [8, 23]. The patchy taxonomic distribution of pinopsin across major vertebrate groups [16, 23, 24, 25, 26] is summarized in Figure 1A.

The expression of pinopsin in amphibians is particularly intriguing in that mRNA is detected in the eye and the pineal complex of anurans [10, 21, 40]. Limited surveys of whole genomes and eye transcriptomes of amphibians failed to find pinopsin in several species of anurans as well as in caecilians [24, 40]. For instance, Boyette and co‐workers [40] found complete or partial pinopsin genes in 19 anuran genomes, but only 31 out of 81 anuran eye transcriptomes contained at least a partial pinopsin mRNA. To obtain a better idea of the evolutionary conservation of pinopsin, we surveyed genomic databases of 95 amphibian species for the presence of the complete coding exons of the pinopsin gene. In total, we analysed four species from four families of Gymnophiona (caecilians), eight species from three families of Caudata (salamanders), and 84 species from 27 families of Anura (frogs and toads) (Figure 1B). A maximum likelihood phylogenetic tree of pinopsin protein displayed a topology that agrees, in general terms, with the one expected from amphibian phylogeny (Figure S1). The pinopsin gene was flanked by the same genes in all groups, indicating conserved synteny (Figure 1C and Figure S2).

Caecilians of the most basal extant group, Rhinatrematidae, have a complete pinopsin gene, while members of the Ichthyophiidae, Dermophiidae and Siphonopidae families lost the gene. In contrast, all salamander genomes surveyed have a complete pinopsin gene. Among anurans, the pinopsin gene could be retrieved from the genomes of 77 species from 25 families, ranging from the basal, paraphyletic Archaeobatrachia (Ascaphidae, Discoglossoidea, Pipoidea, Pelobatoidea) to the superfamilies Hyloidea and Ranoidea (Figure 1B and Figure S1). We could only confirm the absence of pinopsin in ranoid anurans of the genus *Gastrophryne* (*G. carolinensis* and *G. elegans*), which belong to the family Microhylidae, and the genus *Ptychadena* (*P. robeensis*, *P. nilotica*, *P. harenna*), from the Ptychadenidae family (Figure 1B). In *G. carolinensis* and *P. robeensis*, pinopsin was missing from chromosome sequences containing the genomic neighbourhood typically found around pinopsin (Figure 1C and Figure S2), indicating that the failure to retrieve pinopsin from these species was not due to an incomplete genomic sequence.

In conclusion, the pinopsin gene underwent multiple independent losses across vertebrate lineages. In amphibians, however, the gene remain conserved in most species and families of salamanders, frogs, and toads. This widespread conservation suggests that the functions attributed to this opsin in *Xenopus laevis* may be broadly relevant to other amphibian taxa and potentially to vertebrates more generally. Therefore, we examined the biological role of pinopsin in *X. laevis* tadpoles at a specific developmental time where physiological responses triggered by the eye and pineal complex are separated.

### 3.2 Melatonin‐Mediated Changes in Light/Dark Pigmentation and Behavior in Stage 46/47 *Xenopus laevis* Tadpoles Are Regulated by a Photosensitive Pineal Complex, Independently of the Eyes

To investigate the biological function of pinopsin, we established a model using *X. laevis* tadpoles in which two light‐sensitive responses were assessed: (1) melatonin level fluctuations, inferred from changes in skin pigmentation, and (2) daily locomotor activity, analyzed through movement patterns over a 24‐h photoperiod (12h light ON/12h light OFF).

**Figure 2:**
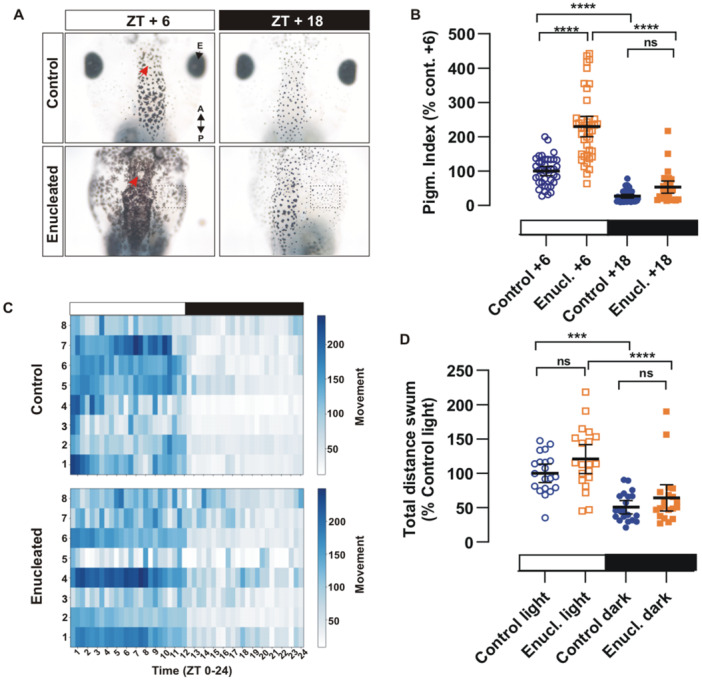
Daily changes in melatonin‐mediated skin pigmentation and locomotor activity are regulated by the pineal complex in stage 46/47 *Xenopus laevis* tadpoles. (A) Skin pigmentation responses to environmental light conditions at ZT+6 (light phase) and ZT+18 (dark phase) in control and enucleated tadpoles. Representative image of the dorsal head views. The pineal complex (red arrow) and a region of increased melanophore density in enucleated tadpoles (square) are indicated. Note that both control and enucleated tadpoles exhibit skin‐lightening in response to melatonin during the night. A, anterior; P, posterior; E, eye (B) Quantification of pigmentation index. Each dot in the panels represents the pigmentation index of an individual tadpole normalized to the control ZT+6 from 3 independent experiments (*N* = 3; *n* ≥ 27). (C) Heatmap of distance traveled over 30‐min windows during a 12 h light ON (white rectangle)/12h light OFF (black rectangle) cycle. The locomotor activity over a 24‐h period for control and enucleated tadpoles is represented, with each row showing data from a separate tadpole. (D) Determination of the total distance traveled during light and dark phases normalized to control in the light phase from three independent experiments (*N* = 3; *n* = 20 tadpoles). In B and D, lines indicate the mean ± 95% confidence interval. Statistical analysis was performed using one‐way ANOVA followed by Tukey's post hoc test. ns, not significant; ***p< 0.001; ****p< 0.0001.

Skin pigmentation in *X. laevis* is governed by melatonin secretion which follows light/dark cycles. Melatonin promotes maximal melanosome aggregation during the night (e.g. Zeitgeber Time [ZT] +18, resulting in peak skin lightening), and maximal dispersion during the light phase (e.g. ZT+6) [29, 30], therefore we chose those ZT times for our further analysis. Of note, in our experimental design temperature (21°C–22°C) and background surface (white) were kept constant, as both factors are known to influence pigmentation responses [17, 41]. Consequently, the only variable was light exposure (light/dark cycles during the zeitgeber period), which, in *Xenopus*, induces pigment change via pineal melatonin secretion [31, 42]. The use of melatonin‐induced skin lightening in *Xenopus* tadpoles as a physiological readout of melatonin levels was pioneered by Lerner and colleagues in 1958 [43], leading to the first biochemical purification of melatonin from pig pineal glands. Soon after, endogenous melatonin released from the adult *Xenopus* pineal complex was shown to trigger physiological skin pigmentation responses [31, 42]. The melatonin response is mediated by the expression of the melatonin receptor 1c in amphibian skin melanophores—a receptor that was lost during evolution in the mammalian lineage [44, 45]. Recent findings from our lab indicate that light sensitivity in the eye and pineal complex are functionally segregated during early larval development (stage 43/44). Indeed, skin lightening still occurs when the pineal complex is obscured with aluminum foil and in enucleated but not pinealectomized tadpoles when the light intensity falls below a threshold [2], confirming the contribution of pineal photoreception to the melatonin‐mediated skin blanching. We wanted to conduct both locomotor and pigmentation studies at a comparable developmental stage, and so decided to use stage 46/47 tadpoles, as movement analysis becomes more reliable at this later stage [21, 46, 47]. Thus, we needed to verify that at stage 46/47 the eye and pineal complex light sensitive responses remain separate. Tadpoles maintained under a 12‐h light/12‐h dark cycle were enucleated at developmental stage 42/43. Two days later, corresponding approximately to stage 46/47, we measured the skin pigmentation index over the head during the middle of the light (ZT+6) and the dark (ZT+18) phases (Figure 2A). As show previously for younger tadpoles [2], enucleated tadpoles were significantly darker than controls (Figure 2A). This eye‐dependent response, known as ‘background adaptation’, is triggered by melanosome dispersal induced by αMSH (alpha melanocyte‐stimulating hormone) secreted from the pars intermedia of the pituitary gland. Notably, enucleated tadpoles behave as if they are on a black surface, even when tadpoles are raised on a white background [48, 49]. The pineal complex remains free of pigmented cells (Figure 2A; red arrow) and the number of pigmented cells increases in the periocular region (Figure 2A; square). This perioptic pigmentation response is phenotypically similar to that previously described in tadpoles adapted to a black background [17, 49, 50]. Nevertheless, melatonin‐induced lightening at night (ZT+18) occurs in both control and enucleated tadpoles (Figure 2A and Figure 2B), suggesting the response is eye‐independent and depends on the photosensitive pineal complex.

Next, we examined locomotor activity over a 24‐h period in stage 46/47 tadpoles. *X. laevis* tadpoles are diurnal [47, 51]. Consistent with this phenotype, the tadpoles exhibit increased locomotor activity during the light phase as compared to the dark phase (Figure 2C and Figure 2D). Remarkably, enucleated tadpoles maintained the same diurnal locomotor pattern as non‐surgical controls (Figure 2C and Figure 2D), indicating that at stage 46/47 the pineal complex serves as the primary photosensor driving the daily light/dark behavior, while the eye plays a minimal role.

Together, these results confirm that the light sensitive pineal complex regulates melatonin release and locomotor movement, which can be read out through skin pigmentation and daily locomotor activity, respectively, setting the conditions for further studies tackling the biological function of pinopsin.

### 3.3 Pinopsin is Co‐Expressed With the Melatonin‐Synthesizing Enzyme Aanat in Cells of the Pineal Complex, and Protein Levels Are Elevated During the Dark Phase

**Figure 3:**
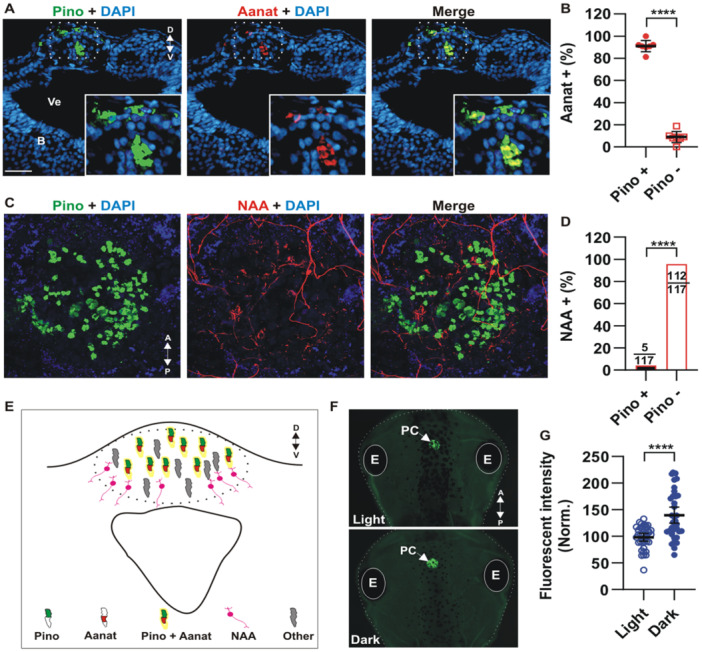
Pinopsin is expressed in melatonin‐synthesizing cells, and its protein levels vary across the light/dark cycle. (A) Immunohistochemistry for pinopsin (Pino; green) and aralkylamine N‐acetyltransferase (Aanat; red) was performed on a transverse section of the brain and pineal complex from stage 46 tadpoles. Nuclei were stained with DAPI (blue). An enlarged view of positive cells in the pineal complex (boxed area) is shown in the bottom right corner. Ve, ventricle; B, Brain; D, dorsal; V, ventral. (B) Quantification of the percentage of Aanat‐positive cells colocalized within pinopsin (*n* = 7 sections from 7 tadpoles; around 92 total pinopsin + cells counted). (C) Dorsal view of the pineal complex of stage 46 tadpoles processed for whole mount immunostaining against pinopsin (Pino; green) and Neurofilament‐Associated Antigen (NAA; red). A Z‐stack confocal image (55 µm depth) is shown, with a 3D image generated with IMARIS software shown in Figure S4. (D) Quantification of the percentage of NAA‐positive cells colocalized with pinopsin (*n* = 3; The number of cells analyzed is shown). (E) Schematic of a transverse section showing the pineal complex. Pinopsin colocalizes with Aanat in the central area, with NAA+ projection neurons located mainly to the periphery. (F) Whole mount immunolabeling of pinopsin in the pineal complex (PC) (white arrow) from a dorsal view from tadpoles fixed at mid‐light (ZT+6; light) and mid‐dark (ZT+18; dark) phases. A, anterior; P, posterior. E, Eye. (G) Quantification of fluorescence intensity in individual tadpoles (dots), normalized to the mean of the light phase (100%) (*N* = 2 experiments; *n* = 36 tadpoles). Statistical analysis was performed using an unpaired t‐test with Welch's correction (****p< 0.0001).

Our earlier work demonstrated that mRNAs for multiple opsins, including *opnp*, *opnpp*, *opnpt*, *opn5*, *opn4*, and *opn4b*, are expressed in the pineal complex [17]. Here, we focused on pinopsin as a possible photosensor for light‐mediated regulation of the tadpole circadian responses, due to its proposed role in regulating circadian rhythms in birds [9, 22, 52]. We developed an antibody targeting the carboxy‐terminal region of *Xenopus laevis* pinopsin and confirmed by immunohistochemistry its localization in eye photoreceptors and the pineal complex. These findings correlate with our previous data obtained via *in situ* hybridization [21]; Figure 3A and Figure S3). We used the pinopsin antibody to investigate whether pinealocytes in non‐mammalian vertebrates can both sense light and synthesize melatonin. Specifically, we analyzed if pinopsin was expressed in the same cells as the melatonin‐synthesis enzyme Aanat [15, 28]. More than 90% of the cells that expressed pinopsin were also Aanat immunopositive (Figure 3A and Figure 3B). In contrast, the projection neurons of the pineal complex, whose axons were labelled with an antibody against Neurofilament Associated Antigen (NAA) [53], did not express pinopsin (Figure 3C, Figure 3D and Figure S4). These data argue that pinopsin‐sensory cells likely both produce and secrete melatonin, communicating with projection neurons that are mainly found in the periphery of the pineal complex (Figure 3C, Figure S4 and schematic in Figure 3E).

In birds, ANNAT is regulated in a circadian manner, showing 7–12 times more activity in the dark than in the light phase [51]. Additionally, pinopsin gene expression is also light‐dependent, regulated by a negative cis‐regulatory element located in the gene's promoter [54, 55]. Pinopsin mRNA levels increase about fivefold during the second half of the light phase followed by troughs during the dark phase [56]. Unknown is whether these changes in mRNA are reflected at the protein level. Therefore, we measured the fluorescence intensity from anti‐pinopsin whole mount immunohistochemistry of the pineal complex of tadpoles fixed at opposite phases of the light/dark cycle (ZT+6 and +18). As expected, pinopsin protein levels were significantly higher in the dark than in the light phase (Figure 3F,G).

Together, these data suggest that pinopsin may regulate the internal circadian cycle by directly influencing melatonin production in pinealocytes, potentially through its colocalization with Aanat. Elevated pinopsin protein levels at night likely facilitate a rapid light‐suppressive response at sunset, aligning melatonin synthesis with environmental light cues.

### 3.4 Pinopsin Regulates Melatonin‐Associated Pigmentation Variation

**Figure 4:**
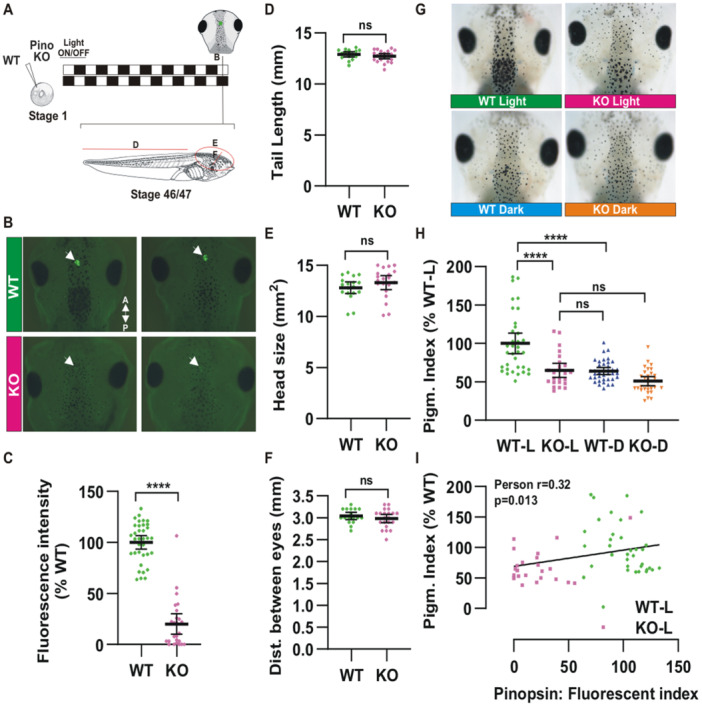
Loss of pinopsin enhances melatonin‐associated lightening of skin pigmentation without affecting tadpole gross morphology. (A) A schematic timeline outlining the experimental procedure, beginning with the generation of CRISPR/Cas9 F0 pinopsin knockout (KO) tadpoles, ending with the phenotypic analysis at developmental stage 46/47. Tadpoles were maintained under a controlled 12‐h light ON/12‐h light OFF cycle. (B) Representative dorsal head images of wild type (WT) and pinopsin KO tadpoles, visualized after whole mount immunostaining against pinopsin. Note the expression of pinopsin in the pineal complex of WT but not KO (white arrows). A, Anterior; P, Posterior (C) Quantification of the fluorescence intensity in the pineal complex. Data are normalized to WT (N3; *n* ≥ 25). (D–F) Tail length (D), head size (E), and distance between eyes (F), comparing WT and KO tadpoles at stage 47. (G) Representative images from the top of the head of WT and KO tadpoles at ZT+6 (light) and ZT+18 (dark). (H) Quantification of the head pigmentation index. Data are normalized to WT (*N* = 4; *n* ≥ 25). (I) Correlation between pinopsin expression determined by fluorescence intensity and the head pigmentation index for the same tadpole. Each dot represents a tadpole (*N* = 3; *n* ≥ 25); The correlation Pearson index, r and the p value are indicated. Statistical significance was determined using an unpaired t‐test with Welch's correction (C–F) or multiple ANOVA followed by Tukey post hoc test (H). In all graphics, each dot represents a tadpole. Lines are mean with 95% confident interval. ns: non‐significant, ****p< 0.0001.

To investigate the role of Pinopsin in pineal complex function and the light/dark response, we generated a loss‐of‐function model in *Xenopus laevis* using CRISPR/Cas9‐mediated gene editing. F0 embryos were produced by microinjecting guide RNAs at the single‐cell stage alongside Cas9 protein. Knock‐down efficiency was assessed by whole mount immunohistochemistry at developmental stage 45–46 approximately 7 days post‐injection (Figure 4A), revealing a marked reduction in pinopsin levels within the pineal complex (Figure 4B). The knock‐down was highly effective, with 90% of injected embryos exhibiting little to no pinopsin immunoreactivity (Figure 4B,C). Consistent with the late onset (stage 39) and spatially restricted expression of pinopsin mRNA during *Xenopus* development [21], general embryonic development and gross morphology, including tail length, head size (area from dorsal pictures), and interocular distance at stage 46, were comparable between wild type and F0 pinopsin‐KO tadpoles (Figure 4D–F). Interestingly, the pinopsin‐KO tadpoles showed a significantly lighter skin in the photo‐phase (measured through the head pigmentation index at ZT+6; Figure 4G), not significantly different from the degree of skin pigmentation in wild type animals from the dark phase (Figure 4H), supporting the idea that pinopsin‐KO tadpoles lose the photic sensitivity necessary to inhibit melatonin synthesis and secretion during the light phase. Importantly, the pigmentation index measured in the tail showed differences comparable to those observed in the head during the light phase (Figure S5), supporting the role of pinopsin in regulating pigmentation through a hormonal mechanism. To determine if pigmentation index is a linear read‐out of melatonin levels, we assessed if the amount of pinopsin expression was correlated with the pigmentation index. Whole mount immunohistochemistry revealed a strong correlation between skin pigmentation and pinopsin expression when analyzed in individual pinopsin‐KO and wild type tadpoles in the photo phase (Figure 4I). Thus, the first light‐sensitive response we assessed, melatonin level fluctuations inferred from changes in skin pigmentation, was clearly disrupted in pinopsin‐KO tadpoles. Moreover, lack of pinopsin alone is sufficient to decrease pigmentation levels during the light phase to levels comparable to those observed under dark conditions.

### 3.5 Loss of Pinopsin Impacts the Locomotor Performance Associated With Light/Dark Behaviour

**Figure 5:**
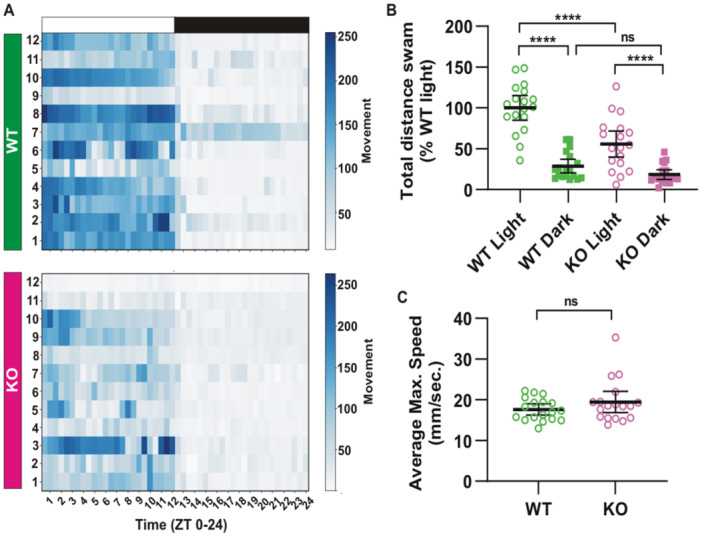
Loss of pinopsin disrupts daily behavior, leading to a reduction in locomotor activity during the light phase. (A) Heatmap plot of distance traveled by wild type and F0 pinopsin KO stage 46/47 tadpoles. Animals were maintained on a cycle of 12h light ON (white rectangle)/12h light OFF (black rectangle). The locomotor activity over a 24‐h period in wild type (WT) and pinopsin Knock‐Out (KO) tadpoles is shown. (B) Total traveled distance in the light phase (L; 12h) and in the dark phase (D; 12h), from WT and KO animals (N = 2; n = 18). (C) Average maximum speed that WT and KO tadpoles reached in the light phase. Statistical significance was determined using multiple ANOVA followed by Tukey's post hoc test (B) and t‐test with Welch's correction (C). Dots represent individual tadpoles, and the lines are mean with 95% confidence interval. ns: non‐significant; ****p< 0.0001.

Next, we examined the second light‐sensitive response, locomotor activity across a 24‐h photoperiod. Like wild type embryos, F0 pinopsin‐KO tadpoles maintained a light/dark response, displaying increased locomotor activity during the light phase as compared to the dark phase, as illustrated by heatmaps showing the average distance traveled every 30 mins (Figure 5A). These data indicate that the KO tadpoles could still recognize photoperiods under a standard 12‐h light ON/12‐h light OFF cycle. The total distance traveled during the light phase, however, was significantly reduced in F0 Pinopsin‐KO embryos as compared to wild type controls (Figure 5B). Despite the decrease in overall activity, both wild type and KO groups exhibited comparable maximum movement speeds (Figure 5C), indicating that motor function itself was not impaired by the loss of pinopsin. Thus, the reduced movement with the loss of pinopsin suggests altered daily behavior, potentially due to unregulated melatonin secretion during the light phase.

Our findings support the hypothesis that reduced daytime movement in pinopsin‐KO embryos results from persistent melatonin signaling rather than motor deficits. Furthermore, the data suggest that pinopsin is not the sole opsin involved in regulating daily locomotor behavior. The partial downregulation of activity and the preserved sensitivity to light/dark phases in pinopsin‐KO tadpoles indicate the likely involvement of additional light‐sensitive opsins in daily locomotor regulation in *Xenopus laevis*.

### 3.6 Stage 47 Tadpoles Lack Circadian Pigmentation and Locomotor Activity Responses

**Figure 6:**
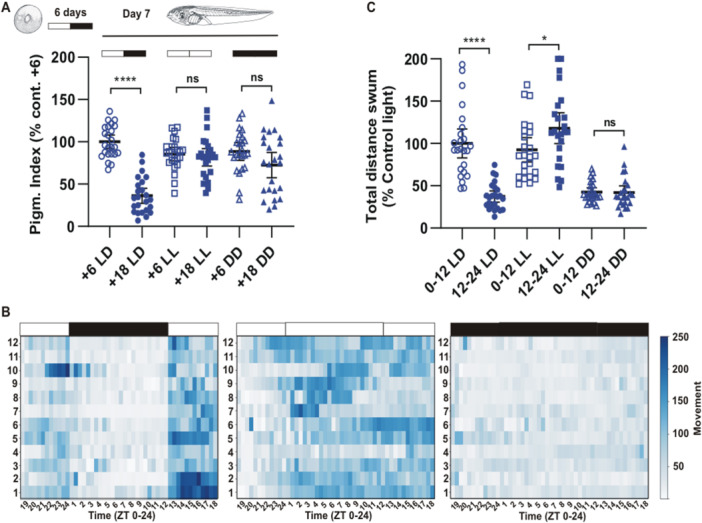
Stage 47 tadpoles lack circadian rhythmic responses. (A) A schematic timeline outlining the experimental procedure of tadpoles raised under a cycle of 12h light ON (white rectangle)/12h light OFF (black rectangle) (LD) from fertilization until day 7 (Stage 47) when larvae were shifted to 24hours of light (LL) or darkness (DD). Head pigmentation index at day 7 measured at ZT+6 and ZT+18 for embryos under LD, LL and DD conditions. Each dot represents the pigmentation index of an individual tadpole normalized to the control ZT+6 from 2 independent experiments (N = 2; n = 24). (B) Heatmap of distance traveled over 30‐min windows on day 7 for tadpoles under LD, LL or DD conditions. The average locomotor activity over a 24‐h period is represented, with each row showing data from a separate tadpole. (C) Determination of the total distance traveled from ZT0 to 12 (light) and ZT12 to 24 (dark) phases for larvae exposed to LD, LL and DD. Data are normalized to control in the light phase from two independent experiments (N=2; n=24 tadpoles). In A and C, lines indicate the mean ± 95% confidence interval. Statistical analysis was performed using one‐way ANOVA followed by Tukey's post hoc test. ns, not significant; *p < 0.05; ****p < 0.0001.

A key unanswered question is whether pinopsin regulates circadian behavioral rhythms in tadpoles. To determine whether an animal exhibits such rhythms, its internal clock must first be synchronized (“entrained”) to an external time cue, like a light–dark cycle. However, previous studies on stage 46–49 tadpoles indicate that, although their pigmentation responses to light–dark transitions are robust [29, 57], the animals themselves do not appear to be entrained, as this response disappears when they are shifted into constant light or constant darkness [29]. We repeated these experiments by measuring the pigmentation index at ZT+6 and ZT+18 of stage 47 tadpoles kept on 12/12 h light ON/OFF cycles from fertilization onwards, followed by a final 24‐h period of constant light or dark (Figure 6A). As expected, the pigmentation level detected for stage 47 tadpoles (day 7) was similar at ZT+6 and +18 when tadpoles were placed under constant light (LL) or dark (DD) conditions (Figure 6A). Notably, pigmentation levels varied considerably for animals placed in constant dark at ZT+18. Several animals showed a punctate pigmentation similar to controls at ZT+18, though the average pigmentation level for the population of larvae in the two conditions remained statistically significant. These data suggest circadian entrainment initiates at a slightly later developmental stage.

Similar results were observed when locomotor activity was analyzed (Figure 6B,C). The heatmap shows that the light/dark–driven pattern of locomotor activity is lost in tadpoles maintained in constant light or constant darkness (Figure 6B), a finding that is also reflected in their total distance swum (Figure 6C). Together, these results indicate that stage 47 wild‑type tadpoles display rhythmic pigmentation changes and locomotor activity in response to environmental light–dark cues. However, they do not exhibit endogenous circadian rhythms in the absence of these cues. This property prevented us from assessing the potential role of pinopsin in circadian regulation. Nevertheless, our findings show that pinopsin contributes to light detection during light/dark responses.

## 4 Discussion

Sensing environmental light for circadian rhythm entrainment requires opsins that become active upon light exposure. Throughout evolution, multiple opsins were selected across vertebrate lineages, giving rise to distinct molecular mechanisms for light detection and circadian regulation. In this study we focus on pinopsin, examining first its evolutionary history across amphibians. Our analysis reveals several, independent loss events of the pinopsin gene within specific amphibian families. Nevertheless, the gene remains largely conserved in anurans and salamanders. We then investigate the physiological role of pinopsin in the pineal complex, by assessing two phenotypic responses: melatonin‐induced skin lightening and daily locomotor activity. Our results show pinopsin plays a significant role in aligning melatonin synthesis with the environmental light‐dark cycle, thereby regulating both physiological and behavioral outputs.

### 4.1 Multiple Independent Gene Loss Events Occurred Across Vertebrate Lineages During the Evolution of Pinopsin

Our genomic survey greatly expands the number of species and families of amphibians where pinopsin has been described. In contrast to our findings, pinopsin was not identified in most species of frogs and toads when scouring eye transcriptomic data [40], perhaps because this opsin is either weakly or not expressed in the retina of most anurans. In our genomic analysis, only representatives of two frog families (Microhylidae and Ptychadenidae) lack pinopsin. Notably, previous surveys have not included salamanders [24, 40], where we found an intact pinopsin gene in the genomes of eight species. Within caecilians, pinopsin is retained in Rhinatrematidae, but lost in representatives of Ichthyophiidae, Dermophiidae, and Siphonopidae. Given the basal position of Rhinatrematidae within Gymnophiona [1, 36], it is plausible that pinopsin was lost only once along the caecilian lineage. However, additional data from a broader range of species and families are necessary to rigorously test this hypothesis. Interestingly, caecilians display several adaptations to a fossorial lifestyle, including a rigid skull, limbless body, and small eyes covered by skin [58]. The widespread absence of pinopsin in caecilians may further support the notion that photosensitivity has diminished in evolutionary importance within this clade.

Overall, pinopsin appears to be broadly conserved among amphibians, although its loss in certain families mirrors patterns reported across major vertebrate lineages. This raises an intriguing evolutionary question: What factors drive the conservation or loss of pinopsin in specific vertebrate groups? Pinopsin was initially discovered in chickens as pineal opsin [9], and has since been proposed to play a distinct role from visual opsins in circadian entrainment to environmental light in birds [12, 56]. An intriguing piece of data in the pinopsin evolutionary history is its absence in crocodilians [23], a close relative of birds within the Archosauria clade. Crocodilians lack a pineal complex entirely, including both the pineal gland and the parietal eye [59]. Nevertheless, they exhibit circadian melatonin rhythms [60], likely produced from extra‐pineal sources such as the Harderian gland or other neuroendocrine tissues [61]. Notably, genes involved in melatonin synthesis, including AANAT and ASMT, are retained in crocodilian eyes and follow a circadian regulation [62]. Interestingly, the pinopsin gene is also lost in mammals [16]. In mammals, the pineal gland lost its light sensitivity and became integrated into the brain, an evolutionary shift that occurred likely during the Mesozoic era, when mammals adopted nocturnal habits to avoid competition with dominant diurnal reptiles [8]. Thus, the loss of pinopsin and the apparent modification or absence of a light‐sensitive pineal complex in both crocodiles and mammals may reflect a broader evolutionary divergence in circadian regulation, with increased reliance on retinal or hypothalamic pathways over pineal photoreception. These data are consistent with our observations in amphibians, in that pinopsin was lost mainly in species adapted to a low light niche (fossorial lifestyle in caecilians). Thus, the anatomical rearrangement of the pineal complex, along with ecological and behavioral differences related to light exposure, may help explain how the evolutionary history of pinopsin was shaped.

### 4.2 Pinopsin is Co‐Expressed With Aanat, and Its Protein Levels Change During the Light/Dark Phases

In pinealocytes, we find pinopsin is co‐expressed with Aanat, the rate‐limiting enzyme in melatonin biosynthesis, suggesting a tightly coupled regulatory mechanism that synchronizes melatonin production with environmental light cues. Pinopsin likely confers photosensitivity to pinealocytes, enabling regulation of both circadian clock function and melatonin synthesis. In birds, Aanat expression is modulated by light exposure at transcriptional, translational, and post‐translational (via phosphorylation) levels [11, 27, 63]. Our results show that pinopsin protein levels are higher comparatively during the night (ZT+18) than during the light phase (ZT+6). These findings are consistent with previous studies at the mRNA level in chicken pineal glands [56]. Pinopsin mRNA expression is partially regulated by cis‐acting elements involved in light‐dependent transcriptional control [55]. Transcript levels remain low during the first half of the day, followed by a sharp increase peaking between ZT+10 and ZT+12, just before the onset of darkness, and then decline during the second half of the scotophase (ZT+18 to ZT+24) [56]. Given that pinopsin is a seven‐transmembrane protein with an uncharacterized turnover rate, a temporal delay between mRNA peak and protein accumulation is anticipated. Collectively, these results suggest that pinopsin is actively transcribed and translated, reaching peak protein levels in preparation for the upcoming light phase. This scenario would allow the organism to effectively sense light and synchronize its internal clock with environmental cues.

### 4.3 Pinospin Regulates Melatonin Mediated Skin Pigmentation and Daily Locomotor Activity

Using F0 CRISPR animals enabled rapid functional screening of pinopsin, though we acknowledge the inherent biological variation introduced by mosaicism. To address this concern, we performed immunostaining on each animal included in the functional analysis to confirm markedly reduced or absent pinopsin protein expression. While the use of F0 animals presents limitations as compared to analyses using stable, homozygous F2 or F3 lines, which require considerable time to generate, our approach offers a fast and robust means of assessing pinopsin function. An additional limitation of our study is the absence of direct melatonin measurements, as skin pigmentation is used as a proxy for melatonin levels. In *Xenopus*, this inference is particularly robust under controlled conditions where the light/dark cycle is the sole variable [17, 31, 42, 64]. Melatonin levels in *Xenopus laevis* were previously quantified using spectrophotometry [65, 66] and radioimmunoassay [67], revealing lower concentrations in animals reared under light compared to those raised in darkness. However, assessing melatonin in F0 pinopsin knock‐out larvae using commercially available systems such as ELISA presents challenges: these assays have not been validated for use in *Xenopus*, and their limited sensitivity may hinder reliable quantification in larval samples.

We identified pinopsin as a key regulator of dark‐associated melatonin synthesis in *Xenopus laevis*, mediating both physiological (skin pigmentation) and behavioral (locomotor activity) responses. However, the dynamics of these two circadian outputs differed. The skin pigmentation phenotype in pinopsin‐KO tadpoles during the scotophase closely resembled that of wild type animals, indicating that pinopsin alone is sufficient to regulate melatonin‐driven pigmentation changes between light and dark phases. In contrast, the persistence of day/light locomotor activity in pinopsin‐KO tadpoles suggests functional redundancy, potentially involving an additional, unidentified opsin(s). Of note, wild type larvae did not exhibit entrainment to drive rhythmic responses, but did show consistent rhythmic patterns when locomotor behavior was monitored over a 48‐h period of light/dark conditions. While total activity increased over time in both wild‐type and KO animals, likely reflecting ongoing developmental progression, KO‐larvae moved consistently less in the photo‐phase than their wild type counterparts.

Melatonin‐induced skin lightening is a well‐established physiological response, historically employed as a bioassay during the early purification of melatonin over 70 years ago [43], with later confirmation that endogenous secreted pineal melatonin induces a circadian regulation of skin pigmentation [17, 31, 42, 64]. Given that pinopsin is expressed in both the eye and the pineal complex, we first confirmed that melatonin‐driven skin lightening depends solely on pineal, rather than ocular, photosensitivity. Our pinopsin‐KO animal model clearly demonstrates that light activation of pinopsin in the pineal complex suppresses melatonin production. This light‐dependent shut‐down mechanism is absent in F0 knockout animals, as evidenced by their complete failure to exhibit skin darkening in response to light exposure.

The reduced locomotor activity observed during the light phase in pinopsin‐KO tadpoles can be attributed to a lethargic response triggered by elevated melatonin levels. Interestingly, this suggests that while pinopsin plays a key role in modulating melatonin‐driven behavioral outputs, the recognition of light and dark phases may involve additional opsin(s). The persistence of phase‐dependent locomotor behavior in KO animals implies that other photoreceptive mechanisms may compensate for the absence of pinopsin in regulating daily light perception. Previous work in our lab demonstrated that the pineal complex expresses multiple opsins [2, 17]. While parietopsin (opnpt) and parapinopsin (opnpp) may play specialized roles in the accessory pineal organs [39, 68, 69], the presence of melanopsin in the tadpole pineal points to a potential contribution to circadian regulation. In mammals, only one melanopsin gene is retained, *OPN4*. *OPN4* is expressed by retinal ganglion cells and plays a critical role in sensing light to regulate the circadian rhythm [3, 4]. Interestingly, OPN4 knock‐out animals partially retain a circadian response in part due to a contribution of signals from photoreceptors directed to the “specialized” intrinsically photosensitive retinal ganglion cells [5, 70]. A homologous melanopsin gene, *opn4b*, is lost in mammals but retained by unknown positive pressure selection in the lineages of several vertebrates, including birds, amphibians, reptiles and fish [19, 40]. Supporting a role of opn4b for correct circadian photoentrainment in non‐mammalian vertebrates, *opn4xa* (homologue of *opn4b*) zebrafish mutants show a modified period length [71]. Like pinopsin, *opn4xa* is expressed in both the eye and the pineal gland [16, 71] Interestingly, *opn4xa* in zebrafish is expressed by the complexes' projection neurons [71]. In amphibian retinas, pinopsin and melanopsin are present in different cells, with the former in photoreceptors and the later in horizontal and retinal ganglion cells [21, 37]. Distinct expression is also likely to occur in the pineal complex. The specific role of eye‐expressed pinopsin, as well as an integrated role of pinopsin and melanopsin in the pineal complex, remain unknown and are currently under investigation.

## 5 Conclusions

Here, we performed a detailed evolutionary analysis of pinopsin in amphibians and determined its physiological function using CRISPR/Cas9 gene editing in *Xenopus laevis* tadpoles. Our evolutionary data indicate that pinopsin is: (1) conserved across most amphibian families, especially anurans (frogs and toads); (2) absent in many caecilians, which inhabit subterranean environments with limited light exposure. These results agree with previous observations across vertebrates in that pinopsin was independently lost in mammals, snakes, crocodiles, and teleost fish, while it is largely retained in birds, turtles, lizards, and non‐teleost Actinopterygii, suggesting a conserved role in light detection.

Our physiological studies in *X. laevis* tadpoles at developmental stage 46/47, when circadian responses rely on pineal rather than ocular light sensitivity, demonstrate that pinopsin: (1) is co‐expressed with Aanat, a key enzyme in melatonin synthesis; (2) suppresses melatonin production during the daylight, as evidenced by paler skin pigmentation in knockout tadpoles; and (3) deficient tadpoles exhibit reduced daytime locomotor activity, consistent with melatonin‐induced lethargy.

Collectively, our findings establish that the pineal complex expresses pinopsin as a critical opsin for light‐regulated behavior in amphibians, particularly *Xenopus laevis*, and support its likely conserved function across non‐mammalian vertebrates such as birds, turtles, lizards, and non‐teleost fish. The interplay among multiple opsins highlights the evolutionary complexity of circadian control mechanisms in these species. Future studies will be essential to elucidate the integrated roles of pinopsin and other opsins within the pineal complex, and to uncover how these photoreceptive systems coordinate physiological and behavioral rhythms.

## Author Contributions

N.H., R.D.R., F.S.J‐deS., and G.E.B. conducted the experiments and analyzed the data. F.S.J‐deS., G.E.B., and S.M. were responsible for experimental design and study supervision. All authors contributed to drafting the manuscript, provided critical intellectual input, and approved the final version.

## Conflicts of Interest

The authors declare no conflicts of interest.

## Supporting information


**Figure S1:** Phylogeny of pinopsin in Lissamphibia.

**Figure S2:** Synteny of the amphibian pinopsin locus.

**Figure S3:** In situ hybridization (ISH) and immunohistochemistry (immuno) against pinopsin mRNA and protein, respectively, show similar labeling in both the eye and pineal complex.

**Figure S4:** Minimal interaction between pinopsin and Neurofilament‐Associated Antigen (NAA) expressing cells.

**Figure S5:** Lightening of skin pigmentation during the light phase (ZT+6) in pinopsin KO tadpoles also occurs in the tail supporting a hormonal regulated mechanism. Quantification of the tail pigmentation index.

**Supplemental Table S1:** Lissamphibian species with a complete pinopsin gene.

**Supplemental Table S2:** Lissamphibian species with well‐assembled genomes missing the pinopsin gene.

## Data Availability

The data that support the findings of this study are available from the corresponding author upon reasonable request.
