# Supplementary figures and images for: Pinopsin Regulates Melatonin Production and Daily Locomotor Activity: Functional Insights From Gene‐Edited Xenopus Tadpoles
Source: J Pineal Res. 2026 Jan 27;78(2):e70114. doi: 10.1111/jpi.70114 (PMC12836464; doi:10.1111/jpi.70114)

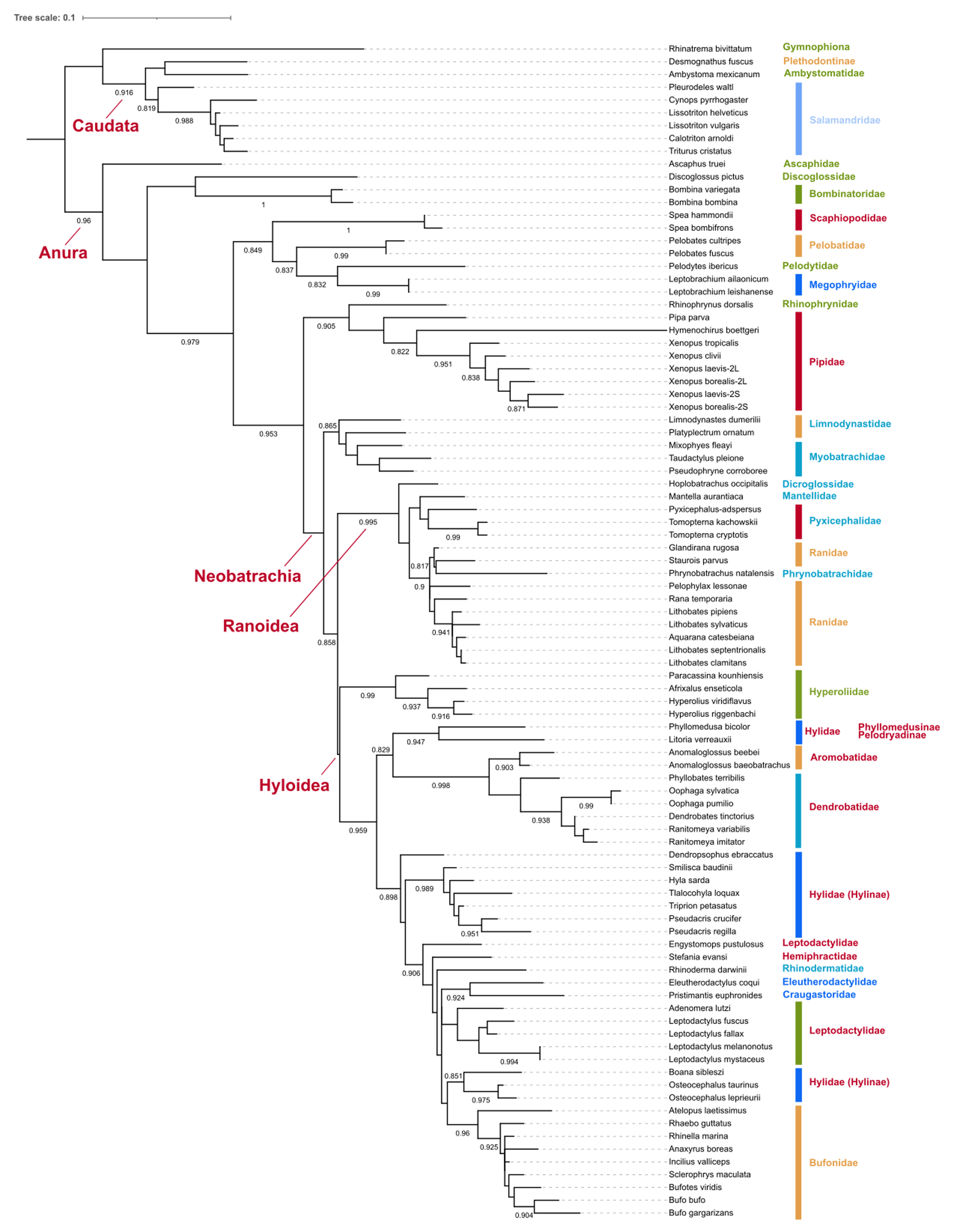

Supplement: Supplementary file 1 — Figure S1: Phylogeny of pinopsin in Lissamphibia. [file JPI-78-e70114-s007.tif]

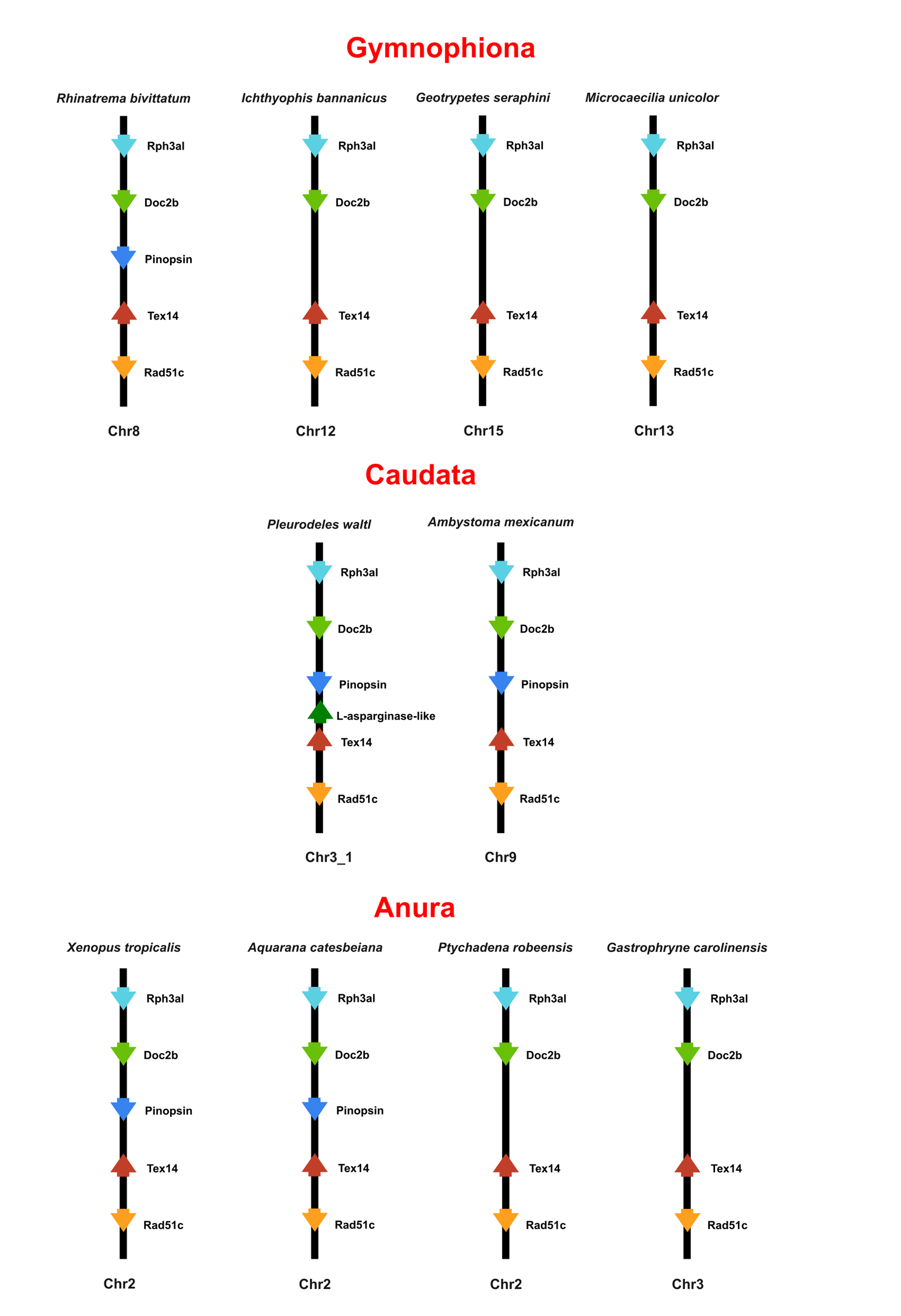

Supplement: Supplementary file 2 — Figure S2: Synteny of the amphibian pinopsin locus. [file JPI-78-e70114-s006.tif]

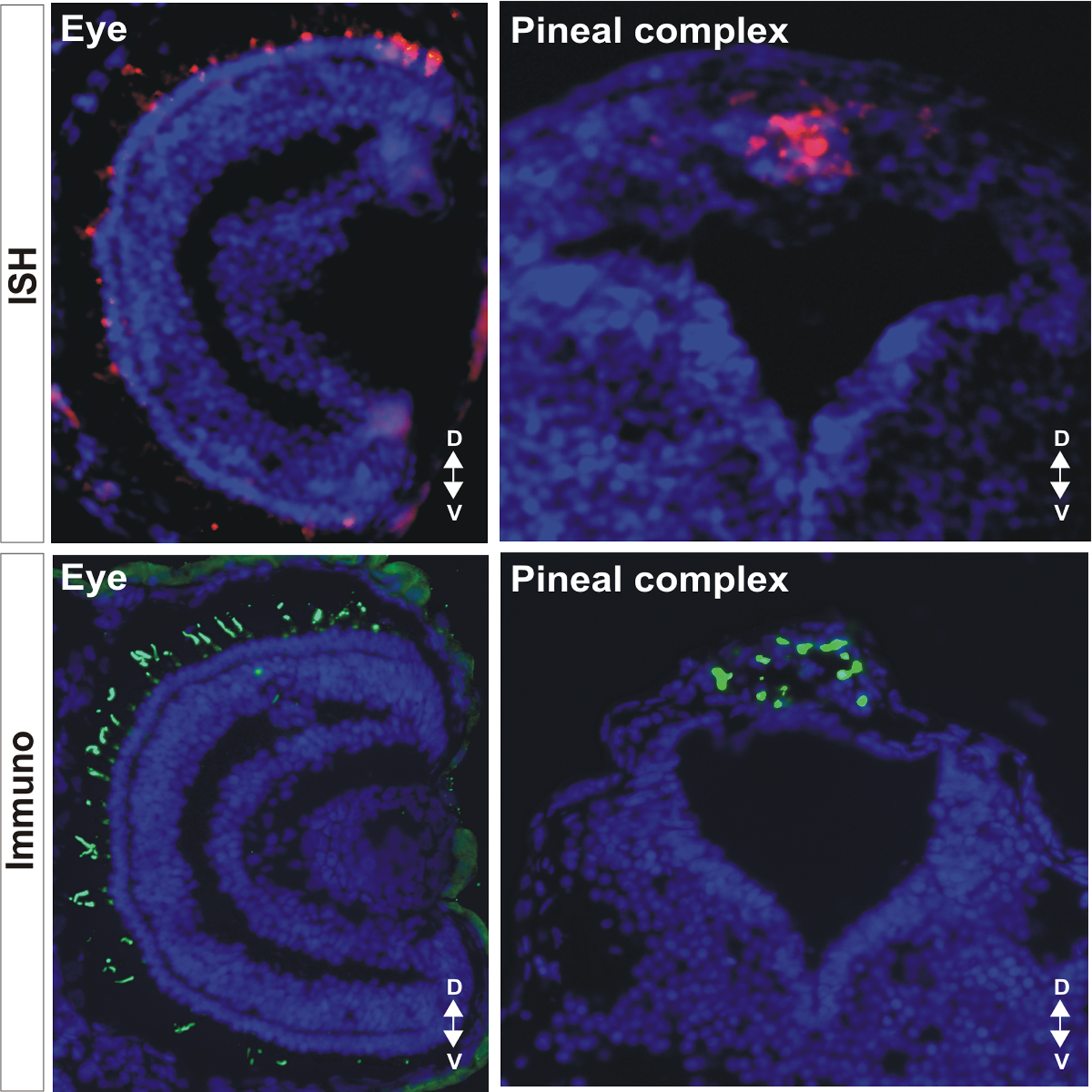

Supplement: Supplementary file 3 — Figure S3: In situ hybridization (ISH) and immunohistochemistry (immuno) against pinopsin mRNA and protein, respectively, show similar labeling in both the eye and pineal complex. [file JPI-78-e70114-s001.TIF]

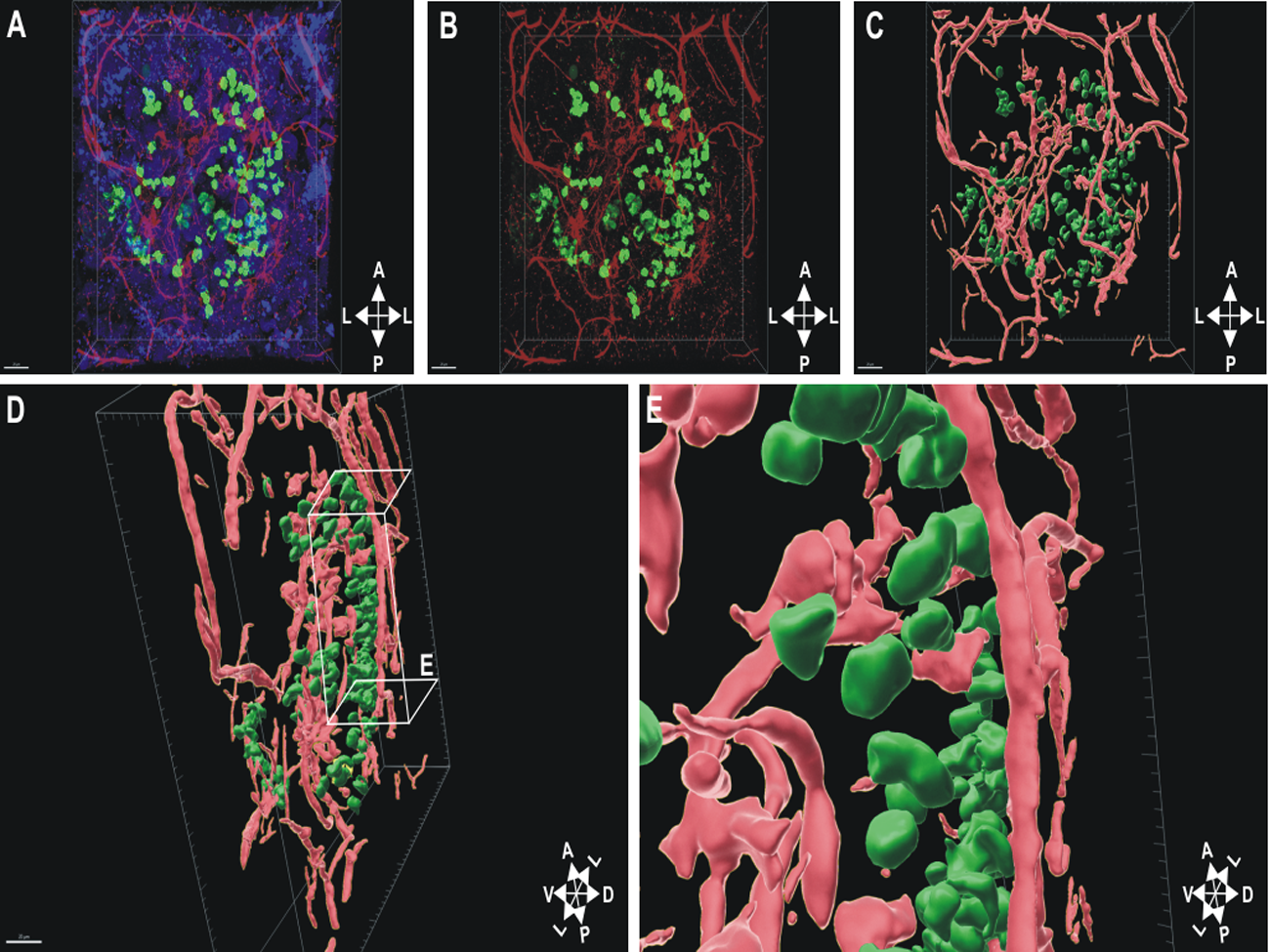

Supplement: Supplementary file 4 — Figure S4: Minimal interaction between pinopsin and Neurofilament‐Associated Antigen (NAA) expressing cells. [file JPI-78-e70114-s003.TIF]

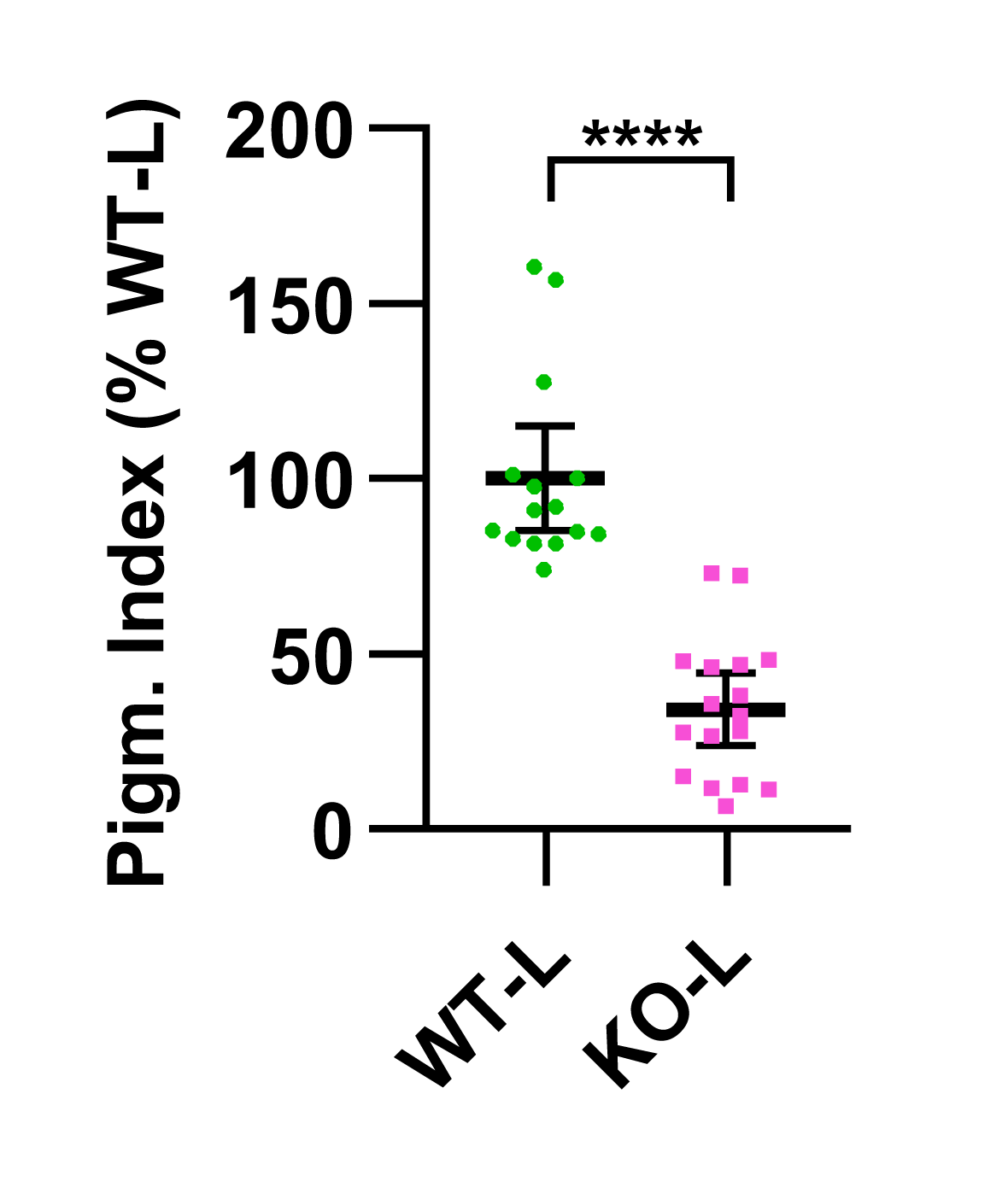

Supplement: Supplementary file 5 — Figure S5: Lightening of skin pigmentation during the light phase (ZT+6) in pinopsin KO tadpoles also occurs in the tail supporting a hormonal regulated mechanism. Quantification of the tail pigmentation index. [file JPI-78-e70114-s004.tif]
